# Draft Genome of *Debaryomyces fabryi* CBS 789^T^, Isolated from a Human Interdigital Mycotic Lesion

**DOI:** 10.1128/genomeA.01580-15

**Published:** 2016-02-04

**Authors:** Hakim Tafer, Katja Sterflinger, Ksenija Lopandic

**Affiliations:** University of Natural Resources and Life Sciences Vienna, VIBT-Extremophile Center, Vienna, Austria

## Abstract

The yeast genus *Debaryomyces* comprises species isolated from various natural habitats, man-made environments, and clinical materials. Here, the draft genome of *D. fabryi* CBS 789^T^, isolated from a human interdigital mycotic lesion, is presented.

## GENOME ANNOUNCEMENT

*Debaryomyces fabryi* is an ascomycetous yeast placed within the family *Debaryomycetaceae* of the order *Saccharomycetales* (1). It was considered for a long time to be a variety of the cryotolerant and halotolerant species *D. hansenii* (2, 3) due to ambiguity of the phenotypic and genotypic boundaries, but on the basis of different fingerprinting techniques and phylogenetic analyses of several protein encoding genes *D. fabryi* has been accepted as separate taxonomic entity (4–7). *D. hansenii* can grow in media containing up to 4 M NaCl and has frequently been isolated from see water, cheese, meat, wine, beer, fruit, and soil, while the origin of the majority of the *D. fabryi* isolates are human skin infections (1). In contrast to *D. hansenii* strains that are able to grow at 31 to 35°C, the maximum growth temperature of the *D. fabryi* strains is 36 to 39°C (2). Recent studies have indicated that *D. fabryi* CBS 789^T^ is more resistant to oxidative stress and more sensitive to fluconazole than *D. hansenii* CBS 767^T^ (8).

In order to determine differences in the genome constitution and gene regulation between two phylogenetically closely related but phenotypically different *D. hansenii* and *D. fabryi* species, the whole-genome sequence of *D. fabryi* CBS 789^T^ originating from an interdigital mycotic lesion was generated. The Ion PI Hi-Q Chef Kit protocol (Life Technologies, Carlsbad, CA, USA) was used to perform emulsion PCR amplification and enrichment of the template ion sphere particles (ISPs). The enriched ISPs were loaded onto Ion PI Chip v3 and sequenced by an Ion Proton semiconductor-based sequencer. A total of 13.7 G with a median read length of 180 bp were generated and assembled with Newbler 2.9 into a 12-Mb genome containing 551 contigs (*N*_50_ 59,311). Ninety-seven percent of the ultraconserved eukaryotic genes were recovered by CEGMA in the genome. Augustus, snap2, scipio, cegma, and glimmer were used to predict protein coding genes. Evidencemodeler summarized the predictions from the various tools into a final protein coding genes set containing 6,027 loci. TRNAscan found all tRNA-isotypes with the exception of SelCys (111 tRNAs). Furthermore, RNAse P, RNAaseMRP, 5 small nuclear RNAs (U1, U2, U4, U5, U6), and 51 snoRNAs were found. Finally two RNA cis-regulatory elements (histone 3′UTR stem loop, TPP riboswitch) were also detected.

### Nucleotide sequence accession numbers.

This whole-genome shotgun project has been deposited in DDBJ/EMBL/GenBank under the accession number LMYN00000000. The version described in this paper is the first version, LMYN01000000.
